# Oligosaccharide prebiotics in functional foods and therapeutics: innovations and challenges

**DOI:** 10.1007/s13205-026-04776-1

**Published:** 2026-06-06

**Authors:** K. S. Sandra, Megh Pravin Vithalkar, Vishnusai Beere, H. B. Bharath, B. Satyanarayana, Mohamed Rafiq, Yogendra Nayak

**Affiliations:** 1 Department of Pharmacology, Manipal College of Pharmaceutical Sciences, Manipal Academy of Higher Education, Madhava Nagar, Manipal, Karnataka 576104 India; 2 Muniyal Institute of Ayurveda Medical Sciences, Manipal, Karnataka 576104 India; 3 Discovery Sciences Group, R&D Centre, Himalaya Wellness Company, Bangalore, India

**Keywords:** Oligosaccharide prebiotics, gut microbiota, metabolic health, functional foods, gut-brain axis, microbiome research, precision nutrition, synbiotics

## Abstract

Oligosaccharide prebiotics, such as inulin, fructooligosaccharides (FOS), and galactooligosaccharides (GOS), have demonstrated significant effects on gut microbiota and host health across in vitro, animal, and clinical studies. These studies consistently report an increase in beneficial bacteria, particularly Bifidobacterium and Lactobacillus, leading to higher production of short-chain fatty acids (SCFAs) such as acetate, propionate, and butyrate. These metabolic changes are linked to improved integrity of the epithelial barrier, reduced inflammatory signaling, modulation of immune responses, and enhanced metabolic balance. Biotechnological production methods, including enzymatic synthesis, microbial fermentation, and controlled depolymerization of plant polysaccharides, allow for precise control over the degree of polymerization and the types of glycosidic linkages. This control directly affects the fermentability, microbial selectivity, and functional effectiveness of the prebiotics. When incorporated into functional food systems, oligosaccharide prebiotics can enhance physicochemical properties such as texture, sweetness, and stability, all while maintaining their biological performance. Advanced delivery technologies, such as nano- and microencapsulation, improve thermal stability, resistance to gastrointestinal degradation, and targeted colon-specific release. Additionally, synbiotic formulations can further enhance the effectiveness of these prebiotics by promoting microbial colonization and sustained availability of SCFAs. Therapeutic benefits have been observed across various models of gastrointestinal health, metabolism, immune responses, and the gut-brain axis. These benefits involve mechanisms such as GPCR activation, histone deacetylase inhibition, and cytokine regulation. However, several challenges remain, including dose-dependent gastrointestinal intolerance, variability in individual microbiomes, degradation during processing, regulatory hurdles, and high costs of downstream processing. Overall, these findings highlight oligosaccharide prebiotics as versatile and scalable biotechnological ingredients, emphasizing the need for standardized production methods, precise dosing, and long-term clinical validation.

**Graphical abstract:**

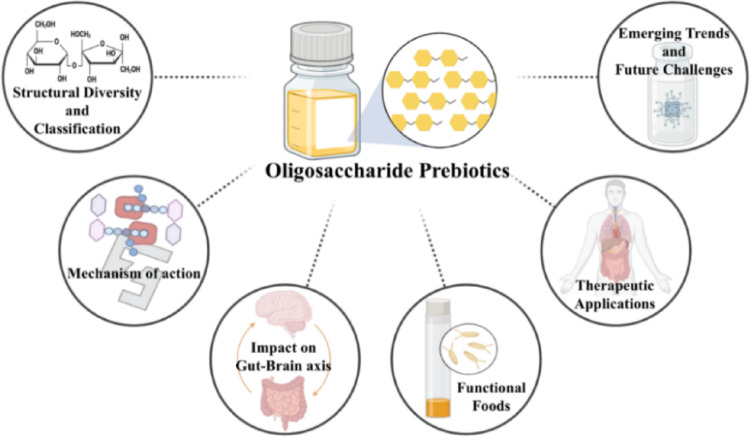

## Introduction

Functional foods are crucial in modern healthcare, offering benefits beyond essential nutrition. Enriched with bioactive compounds, they help prevent chronic diseases and promote overall well-being (Topolska et al. 2021). Recent changes in food consciousness have led us to invest in functional foods, including prebiotics, probiotics, and synbiotics (Al-Habsi et al. 2024). The research area extends to postbiotics, gut microbiome, and further genomic studies such as nutrigenomics and nutrigenetics (Lagoumintzis and Patrinos 2023). Probiotics are food supplements generally containing living bacteria, such as yoghurt, whereas prebiotics are typically foods having inert fibres, such as fruits, vegetables, and grains (Marco et al. 2021). Prebiotics, a key component of functional foods, are nondigestible ingredients that selectively stimulate beneficial gut microorganisms, such as bifidobacteria and lactic acid bacteria (Victoria Obayomi et al. 2024). They are naturally present in plant sources like chicory, onion, garlic, bananas, tomato, corn starch/cobs, apple peels, citrus fruits, sugar beet pulp, yeast cell walls, bamboo, birchwood, oats, barley, soybeans, crustacean shells, asparagus, mushrooms, chitin, konjac root, agave plant, artichoke, cereal brans, brown algae, larch tree, psyllium husk etc. (Chowdhury et al. 2015). They can also be produced using microorganisms and their enzymes through fermentation processes (Lockyer and Stanner 2019). Prebiotics are classified as natural or synthetic, but are generally grouped based on their resistance to digestion, fermentation by the gut microbiota, and health benefits. The general class of prebiotics includes oligosaccharides, polyols, and dietary fibers (Davani-Davari et al. 2019). Prebiotics are characterised by three key attributes: (a) their ability to withstand acidity, enzymatic hydrolysis by the host, and gastro-intestinal absorption; (b) their capacity to undergo fermentation by the gut microbiota; and (c) their selective promotion of the growth and/or metabolic activity of beneficial intestinal bacteria that contribute to host health and well-being (Gibson et al. 2004). Among these, oligosaccharide prebiotics have gained attention for their ability to modulate gut microbiota, enhance immune function, and support metabolic health (Zeng et al. 2023). Oligosaccharides, prebiotics found naturally or synthesised, such as fructooligosaccharides (FOS), galactooligosaccharides (GOS), xylooligosaccharides (XOS), and others, serve as key prebiotic ingredients in functional foods (Table 1). Homogenised samples of oligosaccharides and glycoconjugates can also be obtained chemically, enzymatically, or by other biological methods for systematic studies (Lv et al. 2023). With the growing applications in functional foods and therapeutics, oligosaccharide-based prebiotics are promising to shape the future of preventive healthcare and nutrition. This review explores the innovations and challenges in utilising oligosaccharide prebiotics in functional foods and therapeutics, highlighting their potential in disease prevention and healthcare advancements.

**Table 1:** Comparison of oligosaccharide prebiotics based on chemical structure

Oligosaccharides	Structural Composition	Degree of Polymerization (DP)	Properties	Plant sources	Synthesis	Reference
**FOS**	Fructose units with β-glycosidic linkages and terminal sucrose	2 to over 15, 3 to 30	Low molecular weight, non-digestible, prebiotic	Chicory root, Jerusalem artichoke, garlic, Onion, rye, banana, agave, barley, garlic, tomato, asparagus, honey, wheat	Enzymatic synthesis using Farnesyltransferase (FTase) and β-fructofuranosidase (FFase), transfructosylation of sucrose by fucosyltransferase (FTases)	(Ibrahim 2021)
**GOS**	Galactose and glucose units ((β-(1→2), β-(1→3), β-(1→4), β-(1→))	2 to 8 (commonly 3)	Low molecular weight	Legumes (e.g., soybeans) and Lactose, Whey	Enzymatic synthesis from lactose, microbial fermentation Trans glycosylation of lactose using β-galactosidase	(Yin et al. 2018)
**β-MOS**	Mannose and galactose units	2 to 5	Low molecular weight	Plant mannans like Konjac, porang	Enzymatic or chemical hydrolysis of glucomannan using endo-β-mannanase	(Jana et al. 2021)
**XOS**	Xylose units, sometimes with additional residues	2 to 6	Low molecular weight	Lignocellulosic materials (corn cobs, straw)	Enzymatic hydrolysis of xylan	(Kawee-Ai et al. 2016)
**AOS**	Polyuronic saccharides	2 to 25	Low molecular weight	Brown seaweed	Hydrolysis, organic synthesis, biosynthesis	(Okolie et al. 2020)
**IMOs**	Glucose units linked by α-(1 → 6) and α-(1 → 4) glycosidic linkage	3 to 9	Low molecular weight	Starch hydrolysates from potato peel	Enzymatic (α-transglucosidase)	(Maurya et al. 2023)
**GnOS**	Glucose units (β-(1→6) linkages)	≥ 3	Low molecular weight	Gentiobiose and sucrose	Enzymatic (dextransucrase)	(Kothari and Goyal 2015)

*FOS* Fructooligosaccharides,* GOS* Galactooligosaccharides,* β-MOS* β-Mannan Oligosaccharides,* XOS* Xylooligosaccharides,* AOS* Alginate Oligosaccharides,* IMOS* Isomalto-Oligosaccharides,* GnOS* Gentiobiose-Derived Oligosaccharides

## Comparative analysis of oligosaccharide prebiotics with other functional ingredients

### Differences between prebiotics, probiotics, and postbiotics

**Fig. 1:**
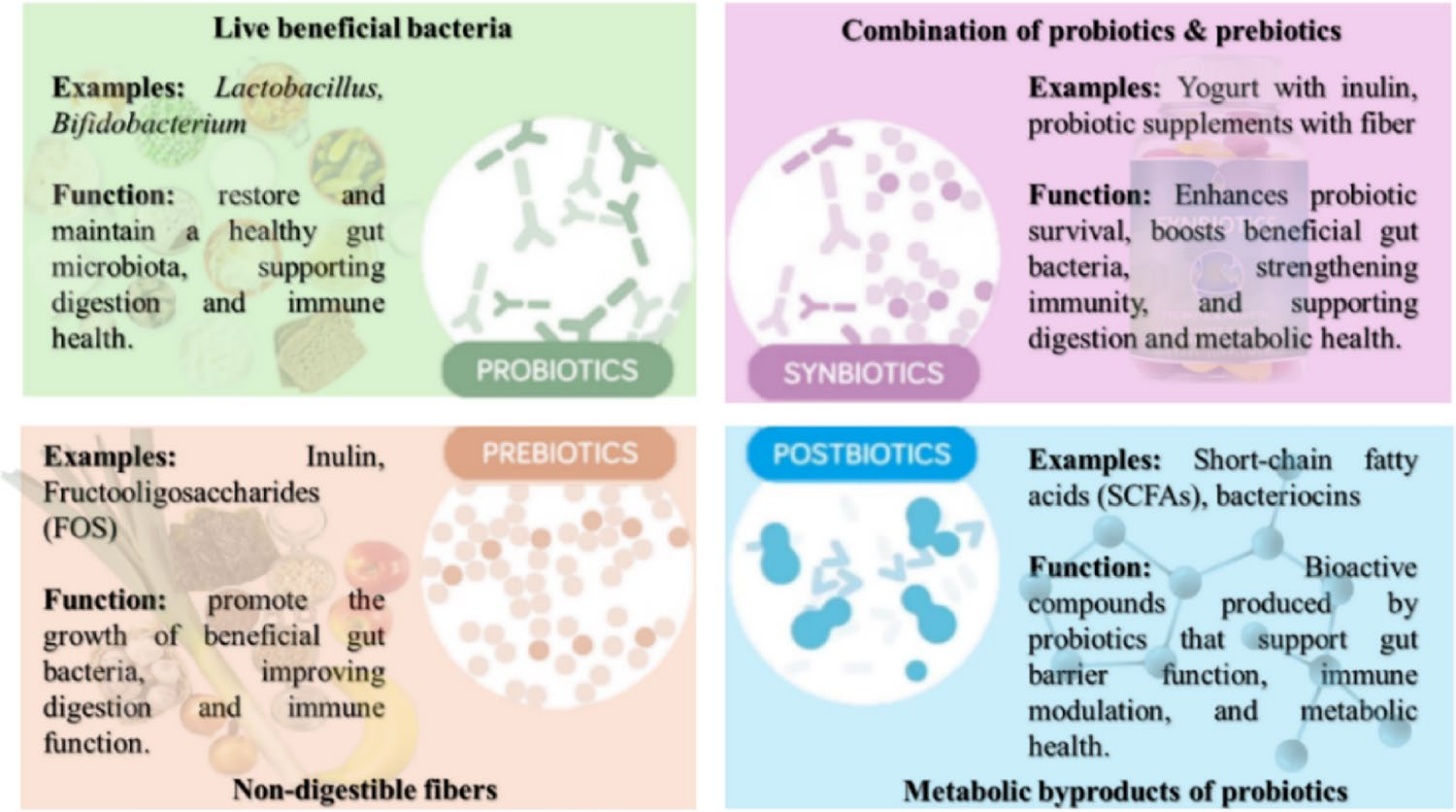
Schematic overview of prebiotics, probiotics, symbiotics, and postbiotics, and their functional distinctions within gut health. Probiotics are live beneficial microorganisms (e.g., Lactobacillus, Bifidobacterium) that restore and maintain a balanced gut microbiota. Prebiotics are non-digestible fibers such as inulin and fructooligosaccharides (FOS) that selectively stimulate the growth of beneficial bacteria. Synbiotics combine probiotics and prebiotics to enhance microbial survival, colonization, and synergistic health effects. Postbiotics represent metabolic byproducts of probiotics, including short-chain fatty acids (SCFAs) and bacteriocins, which exert bioactive effects on gut barrier integrity, immune modulation, and metabolic function

Probiotics are live microorganisms that act through various means, including competing for colonization sites and nutrients, inhibiting growth by producing SCFAs and bacteriocins, modulating the immune response, and improving gut barrier integrity. Examples of probiotics include yogurt, kefir, sauerkraut, kimchi, and miso. Prebiotics are non-living, specialised plant fibers that are selectively utilised by host microbes to alter the microbiome’s composition and activity, promoting reproduction and metabolism of intestinal probiotics. Examples of prebiotics can be bananas, onions, garlic, asparagus, and chicory root. Postbiotics are inactivated bacteria and bacterial components, including cell structures, secretory molecules, and metabolites, which play a vital role in restoring intestinal flora and improving blood glucose levels, exerting a wide range of actions. Examples of postbiotics include SCFAs such as butyrate, amino acids, vitamins B and K, and antimicrobial peptides (Collado et al. 2019). The differences between these terminologies are illustrated in Fig. 1, and a comparative overview is outlined in Table 2.

**Table 2:** Comparative overview of Biotics

Category	Definition	Components and Sources	Mechanism of Action	Health Benefits	Stability and Safety
**Probiotics**	Live microorganisms that confer health benefits when administered in adequate amounts (Chaudhari and Dwivedi 2022)	Beneficial bacteria such as *Lactobacillus* and *Bifidobacterium* (Zaid et al. 2025); found in yogurt, kefir, sauerkraut, kimchi, and miso (Collado et al. 2019)	Colonize the gut, compete for nutrients and adhesion sites, produce SCFAs and bacteriocins, modulate immune responses, and improve gut barrier integrity (Lunder 2015)	Improve gut health, treat diarrhea, reduce IBS symptoms, enhance immunity (Zawistowska-Rojek and Tyski 2022)	Less stable due to requirement for viable organisms; potential adverse effects in immunocompromised individuals (Vallianou et al. 2020)
**Prebiotics**	Nondigestible food components that promote the growth or activity of beneficial microorganisms (Chaudhari and Dwivedi 2022)	Indigestible carbohydrates such as inulin and fructooligosaccharides (Zaid et al. 2025); found in bananas, onions, garlic, asparagus, and chicory root (Collado et al. 2019)	Selectively utilized by host microbes; serve as substrates for probiotics, enhancing their growth and metabolic activity (Chaudhari and Dwivedi 2022)	Improve digestion, enhance gut microbiota composition, and boost immune function (Zawistowska-Rojek and Tyski 2022)	More stable than probiotics; generally safe with minimal side effects (Vallianou et al. 2020)
**Synbiotics**	Combination of probiotics and prebiotics that work synergistically (Chaudhari and Dwivedi 2022)	Probiotics (beneficial bacteria) plus prebiotics (nondigestible carbohydrates) (Parhi et al. 2024) (Collado et al. 2019)	Enhance survival, colonization, and metabolic activity of probiotics in the gut (Singh et al. 2024a)	Improve gut health, enhance immune function, and treat gastrointestinal disorders (Mude et al. 2024)	Stability depends on formulation; generally safe with minimal side effects (Vallianou et al. 2020)
**Postbiotics**	Metabolites and cell-wall components released by live bacteria or after bacterial lysis (Chaudhari and Dwivedi 2022)	Short-chain fatty acids (e.g., butyrate), bacteriocins, amino acids, vitamins B and K, antimicrobial peptides (Xie et al. 2024) (Collado et al. 2019)	Exert anti-inflammatory, antibacterial, immune-modulating effects; restore intestinal flora and improve metabolic parameters such as blood glucose (Mishra et al. 2024)	Enhance gut health, boost immunity, reduce inflammation (Xie et al. 2024)	More stable and safer than probiotics; no need for live bacteria (Vallianou et al. 2020)

###  Synergistic effects of prebiotics with other bioactive

Synergistic combinations of prebiotics with probiotics, postbiotics, and other bioactive compounds enhance modulation of the gut microbiota beyond the effects of individual components. These interactions promote selective enrichment of beneficial microbes, improve intestinal barrier integrity, and strengthen immune regulation by increasing the production of short-chain fatty acids and bioactive metabolites. Clinical and preclinical studies indicate that such combinations provide superior outcomes in gastrointestinal disorders, including irritable bowel syndrome, as well as metabolic dysregulation, demonstrating improved symptom relief and functional efficacy compared with monotherapies (Kumari et al. 2024). Table 3 highlights the combination and its health benefits.

**Table 3:** Oligosaccharide prebiotic combinations and its health effects

Combination	Example/s	Effects	Underlying Mechanisms	References
Oligosaccharides+ Probiotics	Xylo-oligosaccharides + B. animalis subsp. Lactis	Induces bifidogenesis, modulates immune markers	Activation of the mitogen-activated protein kinase (MAPK) signaling pathway, upregulation of TNF-α, IL-1β, COX-2, and iNOS genes, and stimulation of extracellular-signal-regulated kinases (ERK) and c-Jun N-terminal kinases (JNK).	(Childs et al. 2014)
	Fructooligosaccharides FOS + Bacillus subtilis	Improves growth, immune functions, and disease resistance	FOS and BS improved RBC count, Hb levels, total protein, globulin levels, leucocyte count, respiratory burst activity, lysozyme activity, and post-challenge survival.	(Pawar et al. 2023)
Polyphenols + Oligosaccharides	Alginate Oligosaccharide (AOS) + Cyanidin-3-O-glucoside (C3G)	Enhanced gut barrier, immune response, beneficial microbiota	Up-regulation of tight junction proteins, increased mucin-2 and β-defensins, secretion of immunoglobulin A and cytokines	(Li et al. 2023a)
	Arabinoxylan Oligosaccharides (AXOS) + Green Tea Polyphenols (GTP)	Reduced fat mass, improved lipid profiles	Regulation of lipid metabolism, counteractive effects on gut microbiota	(Liu et al. 2024a)
	Cranberry extract (CRX) and isomalto-oligosaccharides (IMOs)	Improved gut health, reduced inflammation, prevented metabolic alterations	Increased butyrate levels, enhanced gut beneficial bacteria, improved gut histology	(Singh et al. 2018)
Oligosaccharides+ Oligosaccharides	fructooligosaccharides (FOS) and galacto-oligosaccharides (GOS)	Anti-inflammatory effects.	Modulating immune responses by enhancing Th1-related and suppressing Th2-related parameters could be beneficial in conditions like allergic asthma.	(Ayechu-Muruzabal et al. 2022)
	lactulose (LAC), raffinose (RAF), and galactooligosaccharides (GOS)	significantly increased the growth of Bifidobacterium breve and Bifidobacterium longum	This combination also increased short-chain fatty acid (SCFA) production, which is beneficial for gut health	(Ehara et al. 2016)
Oligosaccharides + Dietary fiber	GI (galacto-oligosaccharides and inulin), PF (polydextrose and insoluble dietary fiber from bran)	Enhances beneficial bacteria growth	Selectively promoted the growth of Bacteroides or Alloprevotella bacteria, increasing diamine oxidase (DAO) and/or trimethylamine N-oxide (TMAO) values, which was detrimental to health.	(Cheng et al. 2017)

## Structural Diversity and Sources of Oligosaccharide Prebiotics

Oligosaccharides exhibit significant structural diversity, directly influencing their biological functions and prebiotic efficacy. Several factors, including molecular size, composition, and degree of polymerisation, influence the prebiotic capacity of oligosaccharides. These structural characteristics can significantly affect their interactions with the gut microbiota, thereby impacting their prebiotic efficacy. One key aspect is the degree of polymerisation (DP), with oligosaccharides typically having a DP of less than 10, which affects their fermentability and selective utilisation by the gut microbiota (Ooi et al. 2022). Their monosaccharide composition varies, including common sugars such as glucose, galactose, and fructose, as well as less common sugars such as rhamnose and arabinose. The specific monosaccharides and their linkages determine the oligosaccharide’s functionality (Liu et al. 2021a). Additionally, linkage types, particularly the configuration of glycosidic bonds (e.g., β1→4, β1→3), play a crucial role in determining resistance to enzymatic digestion and subsequent microbial metabolism (Zeng et al. 2023). The conformation of oligosaccharides, influenced by factors such as hydroxyl group orientation and ring puckering, shapes their global conformational dynamics (Yang et al. 2023). The molecular size of oligosaccharides, particularly those smaller than 3 kDa, often correlates with a higher prebiotic index due to enhanced fermentation by specific probiotic strains, further highlighting the importance of their structural attributes in modulating gut health (Yoo et al. 2024).

Natural oligosaccharides are highly regarded for their nutritional benefits and complex biological activities, contributing to gut health and overall well-being. In contrast, synthetic oligosaccharides provide precise structural control and enhanced versatility, making them valuable for advanced technological and research applications. A clear understanding of the differences in their production, structure, and functionality is essential for selecting the most suitable type based on specific requirements (Chen et al. 2024b). Table 4 compiles various oligosaccharide prebiotics, their sources, and their reported therapeutic benefits, as reported in the literature.

**Table 4:**
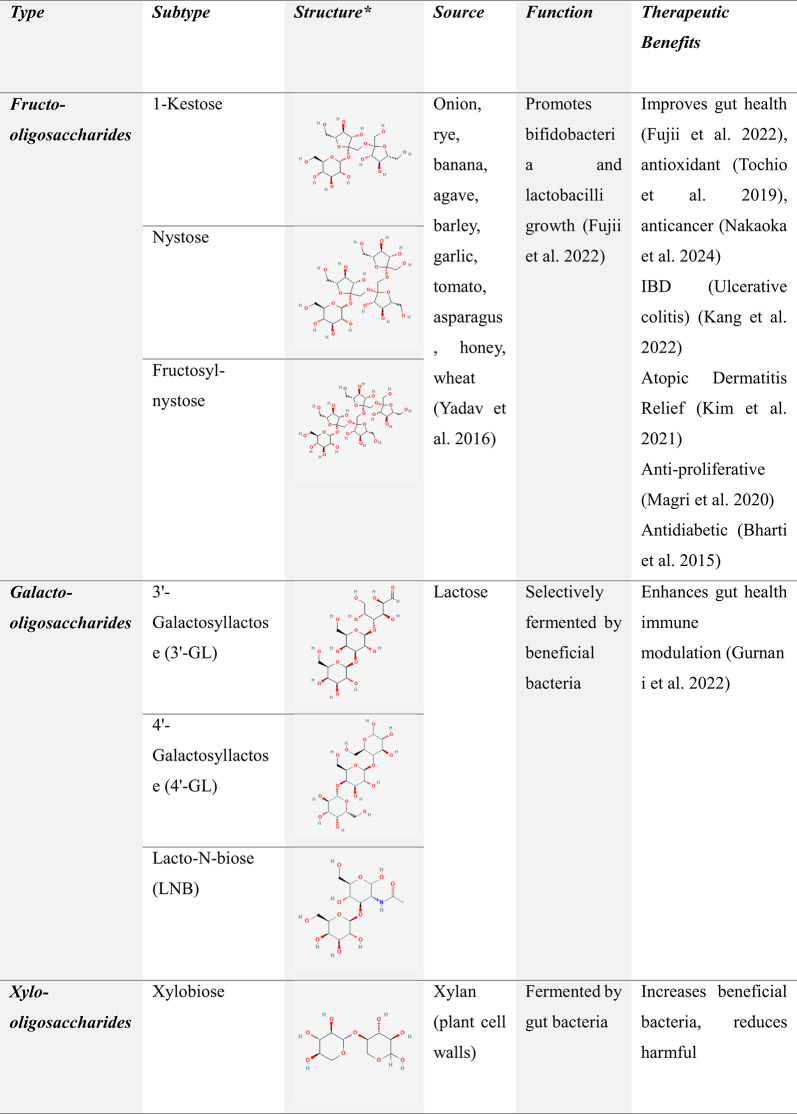

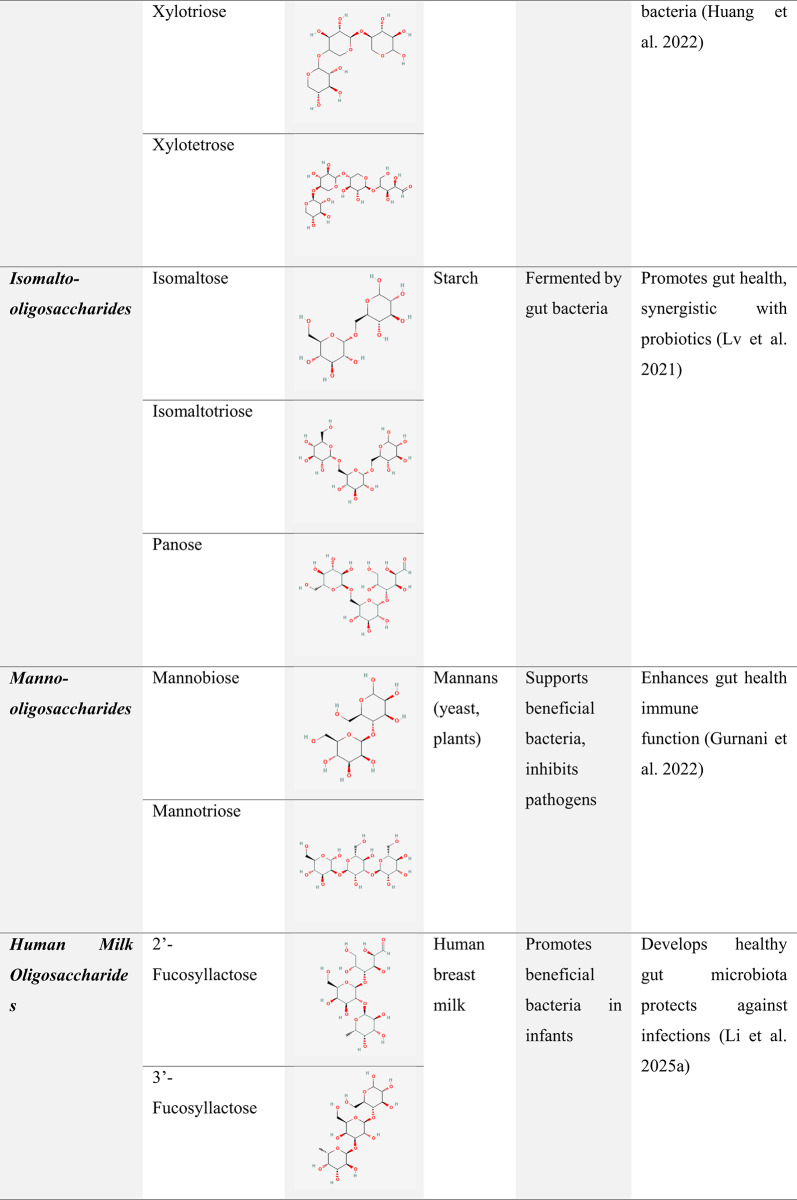

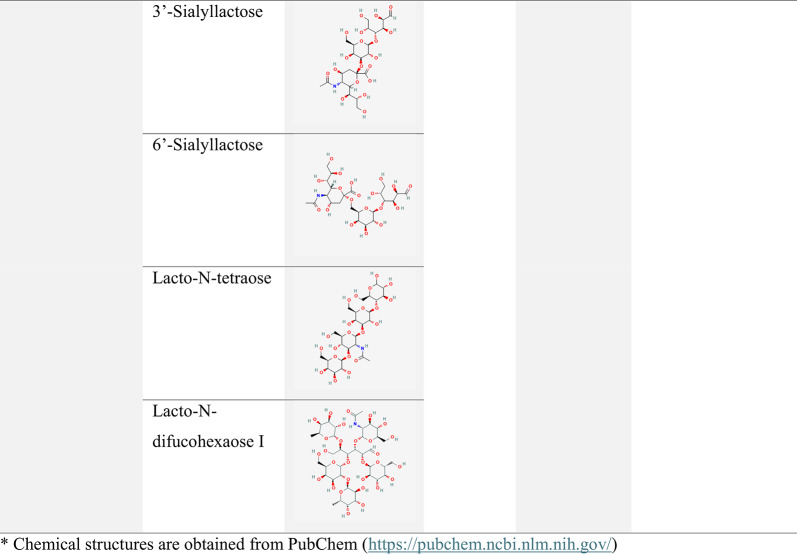
Oligosaccharide prebiotics: Structure, Sources, Functions, and Therapeutic Benefits

## Mechanisms of action of oligosaccharide prebiotics

Oligosaccharides selectively promote the growth of beneficial gut bacteria, including Lactobacillus and Bifidobacterium (Sulek et al. 2014). The fermentation of oligosaccharides by gut bacteria produces SCFAs, such as acetate, propionate, and butyrate, which are crucial for gut health. These SCFAs lower gut pH, maintaining a healthy gut environment and preventing the growth of harmful bacteria (Kazlauskaite et al. 2022). SCFAs help maintain gut homeostasis by maintaining luminal pH, enhancing mucus secretion, and giving energy to colonocytes (Fusco et al. 2023). Recent studies show their function in gene expression, cell proliferation, cell differentiation, apoptosis, and inflammation (Chen et al. 2021). Non-digestible oligosaccharides are certain kinds of oligosaccharides that are resistant to hydrolysis and absorption in the upper gastrointestinal tract. However, gut bacteria can ferment these oligosaccharides in the large intestine, producing SCFAs (Duan et al. 2023).

**Fig. 2:**
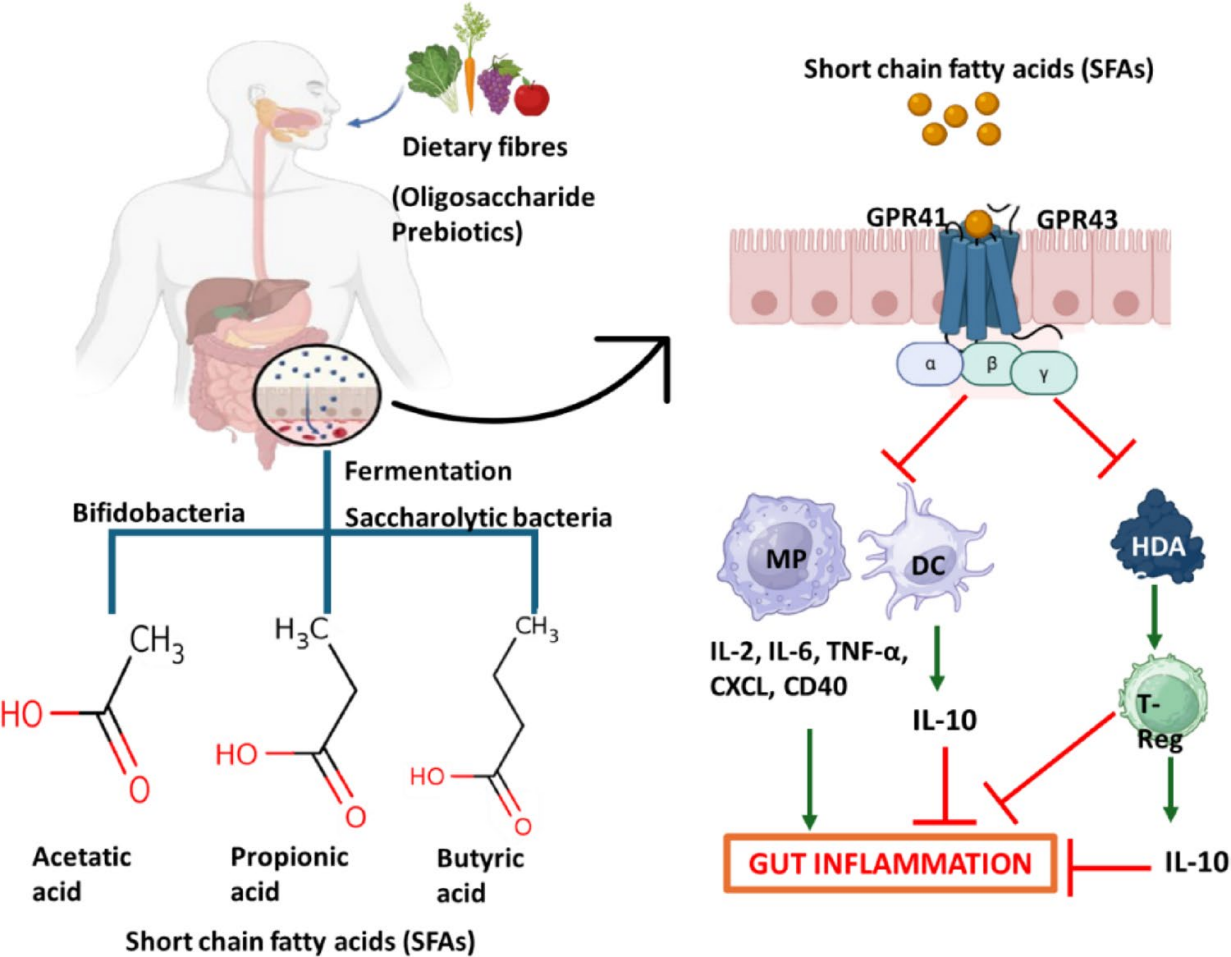
Mechanistic interplay between dietary prebiotics, microbiota-derived postbiotics, and host immune modulation in gut inflammation. Dietary fibers (prebiotics) are metabolized by intestinal microbiota into short-chain fatty acids (SCFAs), including acetate, propionate, and butyrate. These postbiotic metabolites enhance epithelial barrier integrity and signal through host receptors and inhibition pathways. SCFAs modulate immune responses by regulating macrophages (MP), dendritic cells (DC), and promoting regulatory T (Treg) cell differentiation, thereby suppressing pro-inflammatory signaling

Specific oligosaccharides, such as FOS, have shown promising results in alleviating colitis symptoms by modulating the gut microbiota and enhancing the production of anti-inflammatory metabolites through activation of the aromatic hydrocarbon receptor(Yang et al. 2024). FOS has been shown to modulate tryptophan metabolism, leading to the production of beneficial metabolites, such as IAA and IPA, which activate the AhR/IL-22 axis and enhance gut health (Yang et al. 2024). In the colon, XOS enhances strain-specific preferences for fermentation by bifidobacteria and other commensals, producing short-chain fatty acids that moderate immune responses and reinforce intestinal barrier integrity, thereby fostering host health (Schropp et al. 2023). FOS has been shown to promote cell proliferation, increase SCFA production, and enhance the inhibition of biofilm formation by pathogenic bacteria, suggesting its potential as a prebiotic to strengthen probiotic effects on the skin (Shao et al. 2025). XOS and β-glucans modulate gut microbiota and SCFA levels in diabetic rats. While XOS improved blood glucose without altering *Lactobacillus* or *Roseburia*, β-glucan increased both bacterial populations and SCFA (Zahara et al. 2024). Figure 2 illustrates the mechanism of action of oligosaccharide prebiotics.

##  Short-chain fatty acid (SCFA) production and impact on gut health

Oligosaccharides reach the colon and are fermented by the gut microbiota to produce SCFAs, including acetate, propionate, and butyrate (Duan et al. 2023). These SCFAs are key metabolic byproducts of dietary fiber fermentation and play crucial roles in gut health, energy metabolism, and immune regulation. The production of SCFAs depends on specific bacterial species, with Bifidobacteria primarily generating acetate, while other saccharolytic bacteria contribute to propionate and butyrate synthesis (Fusco et al. 2023). SCFAs significantly contribute to colonic health by exerting anti-inflammatory effects, enhancing gut barrier integrity, providing energy for colonocytes, and modulating gut pH, thereby influencing microbial composition. Their mechanisms of action include activating G-protein-coupled receptors (GPCRs) such as GPR41 and GPR43, which regulate immune responses and metabolic homeostasis, and inhibiting histone deacetylases (HDACs), leading to changes in gene expression that support gut health (Wang et al. 2024 b). Beyond the gut, SCFAs impact systemic health through gut-organ axes, influencing the liver, brain, and cardiovascular system while contributing to glucose regulation, energy metabolism, and immune modulation. Increasing colonic propionate production with SCFAs has been suggested as an approach to reduce liver fat in NAFLD patients (Chambers et al. 2019). SCFAs impact neuroimmune signalling by modulating the production of neurotropic factors and reducing neuroinflammation. For instance, a high-fiber diet increases SCFA levels, which correlates with decreased hippocampal inflammation and increased brain-derived neurotrophic factor (BDNF) levels (Church et al. 2023). Their therapeutic potential extends to conditions such as inflammatory bowel disease (IBD), where they have shown promise in treating ulcerative colitis. A study on DSS-treated C57BL/6J mice demonstrated that gavage with SCFA-producing bacterial strains, *Bifidobacterium bifidum H3-R2*,* Propionibacterium freudenreichii B1*, and *Clostridium butyricum C1-6* led to significant improvements in colonic health, reduced inflammatory markers, and altered gut microbiome composition (Yang et al. 2022). Understanding this connection between prebiotic fermentation and SCFA production is essential for leveraging oligosaccharides in functional foods and therapeutics aimed at promoting overall health.

## Influence on gut-brain axis, immune modulation, and anti-inflammatory properties

Oligosaccharides exhibit anti-inflammatory properties by improving gut barrier integrity and reducing pro-inflammatory cytokines. For example, carrageenan oligosaccharides (COS) prevent intestinal inflammation and maintain gut morphology (Wang et al. 2024). HMOs also reduce inflammation in conditions like asthma by modulating gut microbiota and SCFA production (Han et al. 2024). DFO has been shown to boost immune responses and modulate gut microbiota, reducing inflammation (Pansai et al. 2020).

Oligosaccharides such as FOS and GOS can modulate the immune system by influencing gut microbiota, which, in turn, affects gut-associated lymphoid tissue and immune responses (Vijayasarathy et al. 2022). Specific HMOs, such as 2′-fucosyllactose (2′-FL) and 6′-sialyllactose (6′-SL), have been shown to reduce allergic airway inflammation and modulate immune responses via gut microbiota-derived SCFAs (Han et al. 2024; Kim et al. 2024b). POS can enhance immune responses by increasing immunoglobulin levels and reducing pro-inflammatory cytokines, thus protecting against viral infections (Sori et al. 2022).

##  Oligosaccharide prebiotics in functional foods

Oligosaccharides, composed of two to ten monosaccharide residues, are extensively used in the food industry due to their nutritional value, organoleptic qualities, and functional properties that benefit human health. The most used oligosaccharides include GOS, FOS, XOS, MOS, IOS, and AOS (Narisetty et al. 2022). MOS is used mainly in animal feed and functional foods to modulate the gut microbiome (Faustino et al. 2024), while IOS (Kumar et al. 2023) and AOS (Liu et al. 2019) are valued for their bioactive properties, including antioxidant and prebiotic effects. Additionally, FOS serves as a low-calorie sweetener, enhancing the taste of various food products (Narisetty et al. 2022).

Oligosaccharides can be synthesized through multiple methods, including chemical synthesis, enzymatic reactions, and depolymerization of polysaccharides (Chen et al. 2024b). Chemical synthesis, involving complex protection and deprotection steps, is less favored for large-scale production due to its complexity (Talens-Perales et al. 2015). Enzymatic synthesis, using enzymes such as glycosyltransferases, transglycosidases, glycosidases, and glucansucrases, is preferred for its specificity and efficiency. Glycosyltransferases catalyze the transfer of sugar moieties to form glycosidic bonds, while transglycosidases and glycosidases are commonly used due to their high specificity (Plou et al. 2007). Depolymerization methods, such as enzymatic and acid hydrolysis, are widely used to break down polysaccharides into oligosaccharides. Enzymatic hydrolysis, using enzymes like alginate lyases, is favored for its reproducibility and lower cost, while acid hydrolysis is less specific (Zhu et al. 2019). Physical methods, including mechanical extraction, are also employed to obtain oligosaccharides from natural sources (de Moura et al. 2015).

In the food industry, oligosaccharides serve a variety of purposes. They are widely used as prebiotics, supporting the growth of beneficial gut bacteria, enhancing gut health, and promoting overall well-being. As low-calorie sweeteners, oligosaccharides such as FOS are used to reduce calorie content while maintaining sweetness. They are also incorporated into functional foods for their health benefits, including immune modulation, cholesterol reduction, and anti-inflammatory properties (Narisetty et al. 2022). Furthermore, oligosaccharides act as stabilizers and bulking agents, improving the texture and stability of food products (Giese et al. 2011).

Despite their numerous benefits, large-scale production of oligosaccharides presents challenges. Hydrolysis of polysaccharides is considered the most effective approach due to its reproducibility and cost-effectiveness. However, downstream processing (DSP) remains a significant obstacle, as efficient methods are needed to isolate oligosaccharides from the reaction mixture. Overcoming these challenges would enable the broader application of oligosaccharides in the food industry (de Moura et al. 2015; Kruschitz and Nidetzky 2020).

The unique physicochemical and physiological properties of oligosaccharides make them valuable in the food, pharmaceutical, and cosmetic industries. One of the primary roles of oligosaccharides in food formulation is texture enhancement. Interactions with starch can significantly influence the pasting and retrogradation behaviors of starch-containing foods. For instance, FOS and other oligosaccharides modify the viscosity and gelatinization properties of starch, thereby improving texture (Woodbury and Mauer 2023). Additionally, oligosaccharides can act as emulsifiers and stabilizers, like polysaccharides, enhancing the consistency and texture of various food products (Nobre et al. 2015).

Oligosaccharides also contribute to the stability of food formulations by extending product shelf life by limiting starch retrogradation, thereby helping maintain the desired texture over time. XOS, for example, has demonstrated this capability (Woodbury and Mauer 2023). Furthermore, specific oligosaccharides possess antioxidant properties that can prevent oxidative degradation, thereby enhancing the stability and quality of food products (Talens-Perales et al. 2015). In terms of sweetness, oligosaccharides serve as effective low-calorie sweeteners. FOS and GOS provide sweetness without the high caloric content of traditional sugars, making them suitable for low-calorie and diabetic-friendly foods (Gänzle 2012). These oligosaccharides also offer prebiotic benefits, supporting gut health by promoting the growth of beneficial bacteria. This dual functionality of sweetness and prebiotic activity makes them highly valuable in the formulation of functional and health-focused foods (Catenza and Donkor 2021).

The functionality of oligosaccharides as low-calorie sweeteners depends heavily on purity. High-purity preparations (≥ 95% oligosaccharides, DP ≥ 3) have minimal mono- and disaccharides, leading to lower sweetness (~ 15–25% of sucrose) but better prebiotic selectivity, mainly benefiting Bifidobacterium and Lactobacillus. In contrast, less purified fractions (< 80% purity) contain residual sugars, increasing sweetness and solubility for food but decreasing prebiotic efficacy due to broader microbial utilization (Zeng et al. 2023). FOS provides higher sweetness (30–50% of sucrose) with a clean, fruity undertone, while GOS offers moderate sweetness (15–30% of sucrose), with lower-DP fractions perceived as sweet and higher-DP structures presenting a starchy-sweet profile. XOS have the lowest sweetness (10–20% of sucrose), with DP 2–3 contributing mild sweetness and DP ≥ 4 linked to sour or starchy notes. These sensory differences influence formulation strategies, positioning FOS as a dual-function sweetener-prebiotic, while GOS and XOS are typically used for texture modification and microbiome-targeted benefits (Martin et al. 2024).

Oligosaccharides are also employed as fat replacers in various food products. By mimicking the mouthfeel and texture of fats, they can effectively reduce the fat content while maintaining desirable sensory properties. This is particularly useful in dairy and bakery products, where oligosaccharides enhance taste and texture while supporting healthier dietary options. Additionally, using oligosaccharides as fat replacers can reduce overall fat content without compromising taste or quality (Catenza and Donkor 2021; Yurova and Ananyeva 2022). Beyond their roles in texture, stability, sweetness, and fat replacement, oligosaccharides provide additional functional benefits. They act as prebiotics, promoting the growth of beneficial gut bacteria and supporting digestive health. Their dietary fiber content supports digestive healthweight management, and blood sugar regulation (Stribling and Ibrahim 2023; Nikolić et al. 2024).

## Therapeutic applications of oligosaccharide prebiotics

### Gastrointestinal health: treatment of constipation, irritable bowel syndrome (IBS), and inflammatory bowel disease (IBD)

Oligosaccharides act as prebiotics, promoting the growth of beneficial gut bacteria, such as bifidobacteria and lactobacilli, which, in turn, produce SCFAs that enhance gut health (He et al. 2021). A combination of GOS and lactulose has effectively alleviated constipation in loperamide-treated rats by increasing faecal weight, water content, and gastrointestinal transit ratio (Han et al. 2016). Another study uses the combination of GOS and wheat peptides (WP) to alleviate constipation by improving intestinal motility, restoring gut barrier function, reducing inflammation, and modulating gut microbiota. The GOS-WP combination enhances fecal output, water content, and small intestinal propulsion while regulating colonic water transport (Shen et al. 2023). COS can alleviate constipation by modulating key metabolic pathways, including sphingolipid, glycerophospholipid, tryptophan, and bile acid metabolism. COS treatment reversed metabolic imbalances and normalized the genes’ expression in these pathways, suggesting a regulatory role in gut and systemic metabolism (Zhang et al. 2022b). A synbiotic yoghurt containing konjac mannan oligosaccharides (KMOS) and *Bifidobacterium animalis* ssp. *lactis* BB12 (BB12) effectively alleviates constipation by enhancing gut motility, regulating neurotransmitter levels, and modulating gut microbiota composition (Li et al. 2021).

A controlled trial compared the effectiveness of a low fermentable oligosaccharides, disaccharides, monosaccharides, and polyols (LFD) diet with traditional dietary advice (TDA) for treating irritable bowel syndrome with diarrhoea (IBS-D) in Chinese patients. While both diets reduced symptoms, LFD provided earlier relief in stool frequency and excessive wind. LFD also altered gut microbiota, reducing carbohydrate-fermenting bacteria and saccharolytic fermentation activity, which correlated with symptom improvement. Patients with high baseline saccharolytic fermentation activity had more significant symptom burden but responded better to LFD (Zhang et al. 2021).

β-Galacto-oligosaccharide (β-GOS) has shown potential as an adjuvant therapy for ulcerative colitis by improving colonic epithelial barrier integrity and increasing NK cell number, thereby reducing inflammation (Gopalakrishnan et al. 2012). Inulin/TFA/SS NPs, when combined with probiotics, effectively treated IBD in a mouse model of colitis induced by DSS and TNBS. This was achieved by reducing inflammation and oxidative stress, repairing the intestinal barrier, and encouraging rapid proliferation of probiotics to reverse gut microbial disorders (Zhang et al. 2025). FOS derived from *Ophiopogon japonicus* (OJO) exhibited protective effects against barrier function injury in *C. elegans*. In colitis mice, OJP and OJO showed noticeable relief effects, with OJO significantly increasing short-chain fatty acid production compared to OJP (Liu et al. 2024c). 2-In mice with colitis caused by dextran sulfate sodium (DSS), fucosyllactose (2’FL), an oligosaccharide found in human milk, reduces ulcerative colitis. Primary findings that 2’FL provided protection were through immune function modulation and TLR4/MAPK/NF-κB signaling pathway inhibition (Chen et al. 2024a).

###  Metabolic disorders and oligosaccharides: impact on obesity, diabetes, and lipid metabolism

A promising treatment for obesity and metabolic syndrome, the COS-EGCG conjugate (CE) lowers obesity by improving lipid metabolism and encouraging white adipose tissue browning. This is achieved by activating sirtuin 1 (Sirt-1), peroxisome proliferator-activated receptor-gamma coactivator (PGC1-α), and uncoupling protein 1 (UCP1) signaling pathway (Tonphu et al. 2025). 2’-Fucosyllactose (2’FL) supplementation reduces HFD-induced obesity and glucose intolerance by modulating intestinal mucus production, mucin glycosylation, and degradation (Paone et al. 2024). 2′-FL alleviates HFD-induced obesity by enhancing thermogenesis via AMPK activation, increasing mitochondrial content, and upregulating UCP1, PRDM16, and PGC-1α (Li et al. 2024a). 2’-Fucosyllactose (2’-FL) promotes white adipose browning by reducing lipid accumulation and upregulating key thermogenic markers (UCP1, PGC1α, PRDM16) via the AMPK pathway, suggesting anti-obesity potential (Chen et al. 2023). XOS and IMO synergistically reduce weight gain and lipid deposition in high-fat diet-fed mice (Li et al. 2024b). XOS modulates gut microbiota, and IMO enhances insulin sensitivity via the PI3K/Akt pathway (Xu et al. 2023). Their combination promotes fatty acid oxidation and increases acetate- and propionate-producing bacteria (Han et al. 2016; Zhang et al. 2022b). XOS inhibited weight gain, reduced fat mass, improved glucose and lipid metabolism, and enhanced spatial learning and memory. It decreased amyloid plaques and neuroinflammation and reversed HFD-induced hippocampal lipid alterations. XOS mitigates HFD-induced cognitive decline by modulating the gut microbiota, including *Faecalibacterium* (Li et al. 2021; Zhang et al. 2021). Polymannuronic acid (PM) improved gut flora balance, reduced *Escherichia* abundance, and enhanced lipid metabolism. It lowered serum cholesterol and cholesterol ester levels in a dose-dependent manner. However, high-dose PM increased colonic inflammation, elevating lipopolysaccharide-binding protein and interleukin-1β (Zhang et al. 2021).

Coadministration of XOS with carbimazole significantly attenuated oxidative stress, promoted adrenal gland cell regeneration, and mitigated the adverse effects of carbimazole on endocrine function, suggesting that XOS could serve as a therapeutic adjunct for minimising endocrine toxicity associated with antithyroid medications (Zhang et al. 2025). 6-mer HA oligosaccharides activate TLR-2, TLR-4, and CD44 signalling pathways, leading to NF-κB activation and increased production of pro-inflammatory cytokines (IL-1β and IL-6). Concurrently, they downregulate thyroid-specific genes, such as Tg and NIS, contributing to thyroid dysfunction (Liu et al. 2024c).

COSs mitigate maternal diabetes-induced neural tube defects NTDs by suppressing oxidative stress and inflammation, inhibiting TXNIP-NLRP3 complex formation, and reducing pyroptosis in neuroepithelial cells (Chen et al. 2024a; Tonphu et al. 2025). By modifying gut microbiota, improving intestinal barrier integrity, and controlling NKG2D/NKG2DL signalling in GDM mice, XOS and Akkermansia muciniphila act synergistically to ameliorate insulin resistance (IR) in GDM patients (Paone et al. 2024; Tonphu et al. 2025).

Genomic and in vitro analyses revealed that high-GOS-cluster strains exhibited enhanced GOS utilization and bile salt tolerance. In diabetic mice, these strains significantly lowered blood glucose levels (18.52–32.01%) by activating FXR/FGF15/GSK3β and FXR/SREBP1/FAS signaling pathways. GOS treatment increased *Bifidobacterium pseudocatenulatum* abundance, particularly high-GOS-cluster strains (32.03%), modulating bile acid metabolism and FXR agonists (Li et al. 2024b; Paone et al. 2024). Neokestose was orally administered to streptozotocin-induced diabetic rats, significantly suppressing PG elevation. Although neokestose exhibited minimal direct glycosidase inhibition, acid hydrolysis revealed its conversion to blastose, suggesting a synergistic effect on glycosidase inhibition (Li et al. 2024a).

Oligosaccharides from *Ophiopogon japonicus* (OOJ) improve glucose and lipid metabolism in T2DM by modulating the IRS-1/PI3K/AKT/GSK-3β pathway, highlighting their potential as functional food ingredients (Chen et al. 2023). XOS improves insulin resistance in gestational diabetes mellitus (GDM) by enhancing *Akkermansia muciniphila* abundance and strengthening the intestinal barrier. XOS reduced fasting glucose, insulin, HOMA-IR, and inflammation while increasing Akt phosphorylation, occludin, and ZO-1 expression in GDM mice (Li et al. 2024b; Teng et al. 2024). FOS from Cynoglossum tubiflorus roots inhibit α-amylase in diabetic rats, reducing blood glucose and improving lipid profiles in a molecular size-dependent manner (Teng et al. 2024).

### Immunomodulatory effects of oligosaccharides: role in allergies, autoimmune diseases, and infections

FOS supplementation improved atopic dermatitis in children by reducing SCORAD, itching, and sleep disturbance while enhancing skin barrier function (Nikolić et al. 2024). Human milk oligosaccharides 2′FL and 3FL modulate allergen-induced immune responses by influencing epithelial and dendritic cell interactions. In vitro, 2′FL enhanced both inflammatory and regulatory T-cell responses, while 3FL suppressed IL13, promoting IL17 and IL10. HMO supplementation enriched bifidobacteria, delayed microbiome maturation, and influenced amino acid degradation and bile acid conjugation, especially in infants starting before three months. These findings suggest HMO may help correct Cow milk protein allergy-associated dysbiosis (He et al. 2021). FOS enhanced intestinal epithelial barrier function by increasing tight junction protein expression and trans-epithelial electrical resistance while reducing serum markers of barrier dysfunction (DAO, D-lactic acid, endotoxin). These findings suggest FOS may help prevent soybean antigen-induced allergies (Han et al. 2016). COS alleviated shrimp allergen-induced allergy in mice by reducing IgE, histamine, and Th2 cytokines while increasing IgG2a and Th1 cytokines (Shen et al. 2023). A synbiotic combination of *Bifidobacterium breve* M16-V and GOS: FOS: Pectin supports lung health by reducing neutrophilic inflammation, improving lung function, and enhancing gut-derived SCFA production, reinforcing the gut-lung axis concept in respiratory diseases (Li et al. 2021).

The synbiotic intervention containing FOS in SLE patients significantly reduced IL-17 A levels and disease activity over two months. This suggests synbiotics may serve as an adjunctive therapy for SLE and axSpA (Li et al. 2021). Oligo-CS shows chondroprotective effects in OA by inhibiting NLRP3-driven inflammation, reducing cartilage degradation, and modulating gut microbiota. This makes oligo-CS a promising therapeutic candidate for OA treatment (Zhang et al. 2025). COS alleviated experimental autoimmune anterior uveitis (EAAU) in rats by reducing inflammation and NF-κB activation. COS decreased leukocyte infiltration, inflammatory mediator expression, and chemotaxis of sensitized lymphocytes, suggesting its potential as a treatment for acute anterior uveitis (Zhang et al. 2025). AOS reduced intestinal Th17 cell proportions and lowered inflammatory cytokine levels in the periphery. 16 S rRNA sequencing revealed that AOS restored gut microbiota balance disrupted by estrogen deficiency. Additionally, metabolomics analysis highlighted changes in bile acid metabolism, particularly upregulation of Th17 differentiation inhibitors such as isoLCA. These findings suggest that AOS may aid as a promising intervention for osteosarcopenia (Liu et al. 2024c).

### Potential role of oligosaccharides in the gut-brain axis and mental health disorders

The prebiotic diet rich in oligosaccharides normalized VPA-induced alterations in male offspring, including repair of vital microbial taxa, intestinal permeability, peripheral immune homeostasis, reduction of neuroinflammation in the cerebellum, and impairments in social behaviour and cognition in rodents (Prince et al. 2024).

Intermittent fasting (IF), especially with IMO (IF + IMO), mitigates HFHF diet-induced cognitive decline by suppressing TLR4/NFκB signaling, oxidative phosphorylation, and neuroinflammation. IF + IMO modulates the gut microbiome, improves short-chain fatty acid production, and improves cognition via microbiota-derived metabolites (Liu et al. 2024b). Laminaripentaose (LPA) mitigates obesity-induced cognitive impairment by modulating the gut-brain axis. LPA reduces weight gain, hippocampal insulin resistance, and neuronal injury while enhancing gut barrier integrity and lowering inflammation. It increases beneficial gut bacteria, including Butyricimonas and Bifidobacterium, and boosts short-chain fatty acid production (Xu et al. 2024b).

HMOs are the first prebiotics encountered by humans and play a significant role in the microbiota-gut-brain axis. They affect gut bacteria, promote brain development, and modulate the immune system, linking gut homeostasis with the central nervous system and promoting postnatal brain development (Al-Khafaji et al. 2020). XOS can reverse cognitive decline associated with gut dysbiosis by restoring gut microbiota and reducing intestinal inflammation, thereby affecting brain function (Sarkar et al. 2022). MAOS can ameliorate colitis and secondary nerve injury by regulating the gut-brain axis by inhibiting the TLR4/MyD88/NF-κB pathway (Lu et al. 2025).

MOS improves cognitive function and alleviates anxiety-like behaviors in AD mice by modulating the gut microbiota-brain axis. It reduces Aβ accumulation, restores redox balance, suppresses neuroinflammation, and regulates the HPA axis. MOS enhances gut barrier integrity, increases beneficial Lactobacillus, and boosts SCFA (butyrate) production, which correlates with improved brain health (Liu et al. 2021b). XOS mitigates cognitive defects induced by a high-fat diet by increasing the gut microbiota, including *Faecalibacterium*. XOS reduces obesity, improves blood sugar and lipid levels, and enhances spatial learning and memory. It reduces amyloid plaque accumulation, increases BDNF levels, and reduces neuroinflammation in the hippocampus. XOS also restores HFD-induced changes in lipid metabolism and increases beneficial gut bacteria (Teng et al. 2024). Morinda officinalis oligosaccharides (MOO) alleviate depression by modulating the gut microbiota-mediated serotonin pathway. MOO enhances tryptophan hydroxylase activity, increasing 5-HTP production while inhibiting its conversion to serotonin (5-HT), leading to 5-HTP accumulation. Absorbed 5-HTP crosses the blood-brain barrier, boosting brain serotonin levels and improving mood (Zhang et al. 2022b). Konjac oligo-glucomannan (KOGM) effectively improves Alzheimer’s disease (AD) by enhancing spatial learning and memory, reducing Aβ accumulation, and inhibiting Tau hyperphosphorylation. KOGM upregulates BDNF/PI3K/AKT signaling and downregulates GSK3β, promoting neuroprotection. It also reshapes gut microbiota, particularly *Alistipes*, increasing SCFA production (Gou et al. 2024).

### Anticancer effects of oligosaccharides

COS, particularly COS-conjugated selenium (COS-Se), has exhibited immune-enhancing and anti-gastric cancer properties without toxicity. COS-Se treatment significantly suppresses gastric adenocarcinoma growth by downregulating CD34, vascular endothelial growth factor (VEGF), and matrix metalloproteinase-9 (MMP-9) in nude mice (Jiang et al. 2021). Additionally, λ-carrageenan oligosaccharides (λ-COS) exhibit potent antitumor activity and immune-boosting effects, particularly when combined with chemotherapeutic agents such as 5-FU. λ-COS enhances macrophage phagocytic activity, increases TNF-α and IFN-γ secretion, and improves the function of the thymus and spleen in the BALB/c rodent model. Moreover, λ-COS inhibits tumor formation from BGC-823 cells by activating the Par-4 signaling pathway, which TNF-α and IFN-γ activate. Notably, the combination of λ-COS with 5-FU not only enhances anti-gastric carcinoma effects but also mitigates 5-FU-induced immunosuppression, emphasizing its potential as an adjuvant therapy in cancer treatment (Tang et al. 2023). Oligo-Fucoidan, a bioactive oligosaccharide from brown seaweed, enhances olaparib efficacy by modulating immune responses and reducing drug resistance. It promotes antitumoral macrophage polarization and, in TNBC models, synergistically inhibits tumor growth and metastasis by downregulating Rad51, PD-L1, EGFR, and AMPK, key players in drug resistance and cancer stemness (Chen et al. 2022). Cyclodextrins (CDs) improve drug solubility and stability, enabling targeted chemotherapy with reduced toxicity. Red wine-derived oligosaccharides, when combined with methotrexate, enhance tumour suppression without harming normal cells, effectively inhibiting Ehrlich tumour growth (Oliveira et al. 2023). Oligosaccharides also serve as drug-delivery platforms, targeting CD44 receptors to enhance drug encapsulation and selectivity (Jia et al. 2024). Notably, quaternized chitosan oligosaccharide (HTCOSC) targets integrin αvβ3, delivering antiangiogenic and chemotherapeutic agents to suppress tumor growth with minimal side effects effectively (Tang et al. 2024).

The antipsychotic drug penfluridol was repurposed for the treatment of cancer, especially against breast cancer, glioblastoma, colorectal cancer, and other malignancies. It has been found to inhibit glycan processing, leading to the accumulation of high-mannose oligosaccharides and activation of T-cell immunity, suggesting its potential use in cancer therapy (Xu et al. 2024a). Penfluridol treatment has been shown to cause an accumulation of high-mannose oligosaccharides, particularly Man-5-7GlcNAc2 glycan structures, due to its direct inhibition of MAN1A1 mannosidase, a key Golgi enzyme involved in N-glycan maturation. This alteration in glycosylation significantly impacts PD-L1 function, disrupting its interaction with programmed cell death protein 1 (PD-1) and thereby enhancing T-cell-mediated tumor immunity. In mouse xenograft and glioma models, penfluridol has demonstrated the ability to improve the antitumor efficacy of anti-PD-L1 antibodies, suggesting potential in cancer immunotherapy (Xu et al. 2024a).

## Recent innovations in oligosaccharide prebiotic research

**Fig. 3:**
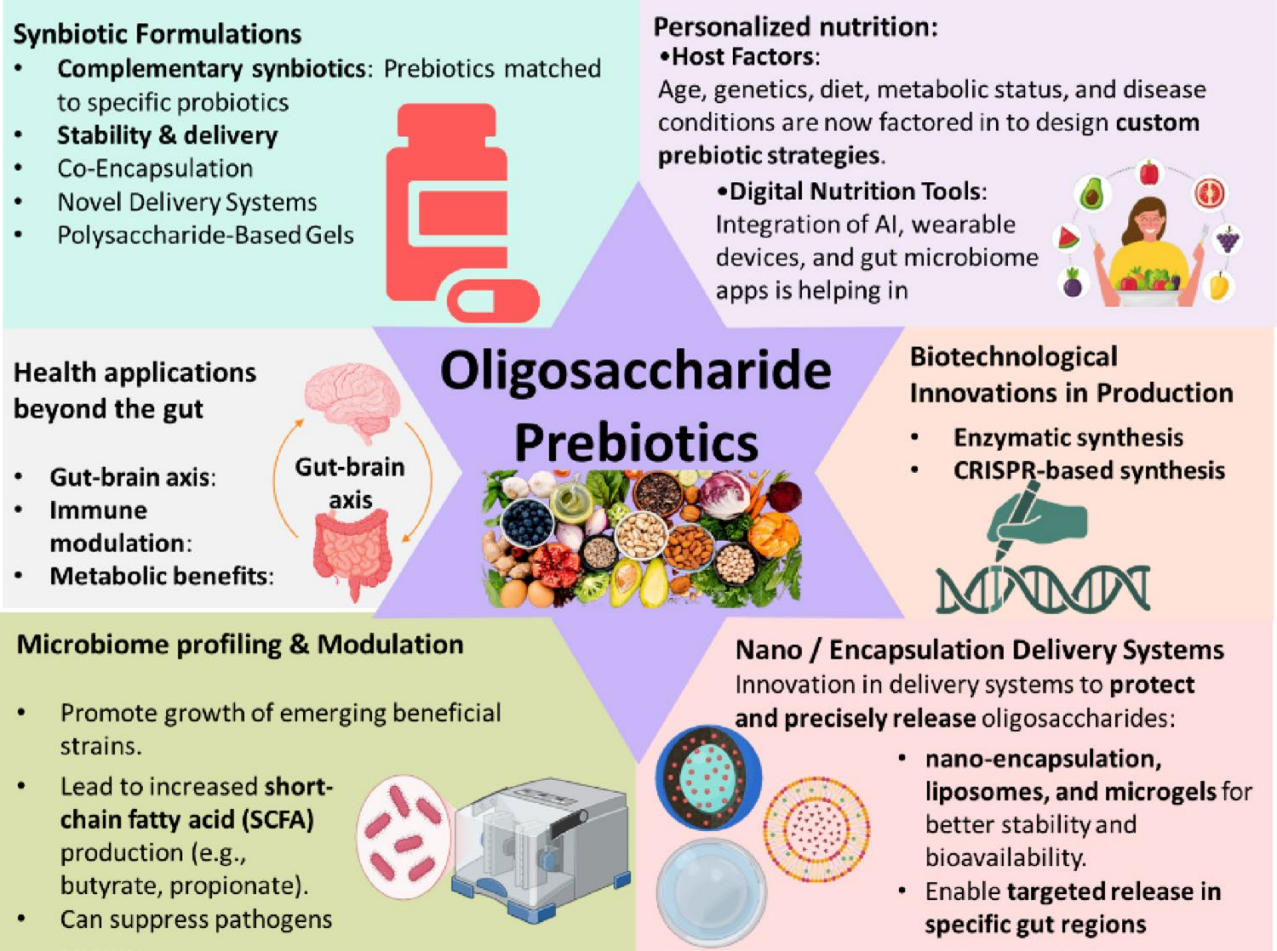
Emerging trends of oligosaccharide prebiotics, including complementary synbiotic formulations, improved stability through co-encapsulation, and novel polysaccharide-based delivery systems. Personalized nutrition approaches integrate host factors (age, genetics, metabolic status) and digital microbiome tools to design tailored prebiotic strategies. Biotechnological innovations such as enzymatic and CRISPR-based synthesis are enhancing scalable production. Advanced nano- and micro-encapsulation systems enable targeted, controlled release within specific gut regions. Beyond gut health, oligosaccharide prebiotics influence the gut-brain axis, immune modulation, metabolic regulation, and microbiome profiling to promote beneficial strains and short-chain fatty acid production

The development of novel oligosaccharides with enhanced prebiotic potential is a growing area of research, given their significant health benefits, particularly in promoting gut health. New bioprocessing methods, including whole-cell and immobilized enzyme processes, have been developed to enhance the production and purification of prebiotics such as FOS and GOS. Research has shown that individual responses to prebiotic interventions can vary significantly, underscoring the need for personalized approaches. Synergistic synbiotics, which combine prebiotics with specific probiotics, offer tailored solutions to enhance gut health. The development of nanoprebiotics, such as whey protein isolate/inulin nano complexes, shows promise for improving the delivery and efficacy of prebiotics. Figure 3 illustrates the emerging trends in research on oligosaccharide prebiotics. Further, the innovations reported are listed below, with descriptions under each subheading.

### Synbiotics: a combination of prebiotics with probiotics for improved efficacy

The combination of probiotics and prebiotics is described as synbiotics (Khursheed et al. 2022). This enhances mutual effects, leading to improved gut microflora, increased mucus production, and the production of anti-inflammatory cytokines and antimicrobial metabolites. Synbiotics have been found to increase levels of anti-inflammatory markers IL-10 and secretory immunoglobulin A (sIgA) in both elderly individuals and healthy adults, indicating an enhanced immune response (Hartono et al. 2018; Lépine and de Vos 2018). In healthy adults, synbiotic supplementation reduced plasma C-reactive protein and interferon-gamma levels, markers of inflammation. This suggests that synbiotics can help in reducing systemic inflammation (Li et al. 2023b). In aquaculture, synbiotics have been shown to enhance disease resistance in fish by improving immune parameters, such as red and white blood cell counts, and increasing survival rates against bacterial infections (Islam et al. 2025). Several studies have shown that synbiotics can improve various IBS symptoms. For instance, a study found that synbiotics significantly improved abdominal pain, defecation symptoms, and psychological well-being in elderly IBS patients (Oh et al. 2023). Another study reported that high-dose synbiotics were superior to placebo in reducing abdominal discomfort, bloating, and fatigue in IBS patients (Lee et al. 2019).

### Application of nanotechnology and microencapsulation for targeted delivery

Nanotechnology-based delivery systems, such as nanoparticles, nanohydrogels, nanoemulsions, and nanofibers, can encapsulate prebiotics to protect them from degradation and facilitate targeted release in the colon. Dual pH-responsive nano-micelles have been developed for the targeted delivery of hydrophobic drugs. These systems can release their payload in response to the pH changes in the target environment, such as the acidic conditions of tumor cells (Wang et al. 2019). Composite nanomaterials, such as ZnO/chitosan oligosaccharide, exhibit effective antimicrobial properties, making them useful for preserving agricultural products and protecting plants (Du et al. 2023). Cyclodextrins are frequently employed to deliver medications and genes in cancer treatment; by precisely targeting these treatments to specific locations, they enhance their anti-proliferative and anti-cancer properties by prolonging their residence time at tumour sites and increasing their circulation time (Lu 2023). Novel amphiphilic oligosaccharides have been developed that stabilize emulsions and provide prebiotic benefits. These oligosaccharides can form nano- and microdroplets, demonstrating exceptional stability and potential for various nutraceutical applications (Li et al. 2025b).

### Emerging research on personalized prebiotic therapy based on microbiome profiling

The effectiveness of prebiotics can vary widely among individuals due to differences in microbiome composition and function. This necessitates personalized approaches to maximize therapeutic benefits. Recent advancements in microbiome science have paved the way for personalized prebiotic therapies, particularly focusing on oligosaccharides. This approach leverages the unique structural characteristics of carbohydrate-based prebiotics (CBPs), including degree of polymerization, branching, and glycosidic linkage, to effectively modulate the gut microbiome (Lam and Cheung 2019). There is significant inter-individual variability in response to prebiotic interventions. Machine learning models have been developed to predict responders and non-responders based on initial gut microbiota profiles, enabling more personalized and effective prebiotic therapies (Kok et al. 2024).

## Challenges and limitations in prebiotic applications

### Stability issues during processing, storage, and digestion

The stability of oligosaccharides varies under different processing conditions. For instance, GOS remains stable during extrusion, while FOS and inulin show significant degradation at higher temperatures (140 °C and 170 °C). Resistant starch (RS) levels, interestingly, increase under these conditions. Similarly, glucomannan oligosaccharides exhibit greater thermal stability than FOS (Ye et al. 2020). In acidic beverages, FOS and inulin are negatively affected, especially at lower pH levels and at specific sucrose-to-corn syrup solids ratios. GOS and RS, however, remain unaffected by these conditions (Duar et al. 2015). Oligosaccharides, such as GOS, can protect probiotics during freeze-drying by immobilizing cells in a glassy matrix, thereby helping maintain cell integrity and viability. This protective effect is crucial for the long-term stability of prebiotics in dried forms. XOS incorporated into emulsion gels shows resistance to lipid oxidation, significantly delaying peroxidation processes during storage. During digestion, oligosaccharides can be partially hydrolyzed, releasing monosaccharides that may alter their prebiotic effects (Lee et al. 2024). For example, galactosylsucrose is slowly hydrolyzed by small intestinal enzymes, which could impact its prebiotic functionality. Microencapsulation using maltodextrin and whey protein isolate can significantly enhance the stability of oligosaccharides, such as Ramulus mori oligosaccharides, by reducing hygroscopicity and improving thermal and storage stability (Zhu et al. 2022). Oligosaccharides such as fructo-oligosaccharides, lactose, and inulin can act as effective lyoprotectants for nanoliposomes, improving their physical stability and reducing cargo leakage during freeze-drying (Jiang et al. 2023).

### Dose-dependent effects: determining optimal consumption levels

There is a notable lack of comprehensive research data supporting the effective and safe dosage of functional oligosaccharides. This gap makes it challenging to establish standardized dosage recommendations for various applications. Determining the appropriate dosage for oligosaccharides can be complex because responses vary across doses. For example, studies have shown that the bifidogenic effects of short-chain fructooligosaccharides (scFOS) vary with dosage, with significant increases in bifidobacteria count observed at doses ranging from 2.5 to 10 g/day (Bouhnik et al. 2006). The effective dosage may vary depending on the specific application and target population. For instance, in animal studies, different oligosaccharides required varying dosages to treat conditions like constipation, with high-dose galacto-oligosaccharides (GOS) being the most effective (Wang et al. 2017). Additionally, studies on *Leuconostoc lactis* CCK940-derived oligosaccharides highlight a dose-dependent increase in mRNA expression of immune-related cytokines, suggesting their potential as immune modulators. In a mouse influenza vaccination model, dietary pectin-derived acidic oligosaccharides (AOS) were found to significantly and dose-dependently improve vaccine-specific delayed-type hypersensitivity (DTH) responses. This enhancement was associated with reduced T-helper2 (Th2) cytokine production, indicating a Th1-skewed immune response (Vos et al. 2006). These variations underscore the need for precise, application-specific dosing guidelines to maximize the health benefits of oligosaccharides while minimizing potential adverse effects.

### Regulatory concerns and labelling: global perspectives on prebiotic claims

The regulation and labelling of oligosaccharide-based prebiotic products remain complex and inconsistent across different regions, posing challenges for researchers, manufacturers, and regulatory authorities. In the United States, the FDA and FTC require rigorous scientific substantiation for health claims, while the European Food Safety Authority (EFSA) enforces stringent clinical evidence requirements, with no prebiotic health claims yet approved. Similarly, regulatory assessments in Brazil highlight frequent labelling inaccuracies, underscoring the need for stricter compliance measures (Thakur et al. 2024). However, the lack of harmonized global regulations complicates market entry and approval, necessitating efforts by organizations like Codex Alimentarius to develop standardized frameworks. Moreover, scientific validation of health claims remains a critical challenge, as regulatory bodies demand robust human clinical trials to demonstrate efficacy, a process hindered by individual microbiome variability and limited long-term studies (Sanders et al. 2019).

Prebiotic oligosaccharides, including human milk oligosaccharides, are subject to safety assessment and, in some cases, novel food approval in several regulatory jurisdictions, including the European Union (EU) and the United States (US). Regulatory oversight in these regions is primarily conducted by the European Food Safety Authority (EFSA) and the Food and Drug Administration (FDA), respectively, with evaluations focusing on toxicological safety, intended use, and manufacturing quality (Salminen 2017). Similar regulatory structures exist elsewhere, such as the Food Standards Australia New Zealand (FSANZ), which governs the approval and use of prebiotics in Australia and New Zealand (Loke et al. 2025). Novel or non-traditional prebiotic ingredients are generally required to undergo rigorous safety evaluations before market authorization to ensure compliance with regional food safety standards (Gallego and Salminen 2016).

The International Scientific Association for Probiotics and Prebiotics (ISAPP) defines prebiotics as “substrates that are selectively utilized by host microorganisms conferring a health benefit”; however, this definition is not uniformly incorporated into regulatory frameworks. As a result, some products labelled as prebiotics may not meet the criteria of selective microbial utilization, leading to inconsistencies across markets. Given the many variables that influence the outcome of prebiotic intake, there may be more to gain from highlighting prebiotic activity as an additional benefit of a specific health effect (Gallego and Salminen 2016).

In the United States, the term prebiotic can be used in structure-function claims if the ingredient qualifies as dietary fiber and does not carry disease-related implications. Labeling must include fiber content, ingredient type, dosage, and potential allergens. Regulatory agencies stress the need for surveillance to ensure accuracy and prevent misleading claims, as the term prebiotic may imply health claims that require scientific backing. (Silva et al. 2010; Brooks and Kalmokoff 2012). The EU takes a restrictive stance on the term “prebiotic,” only allowing it on product labels if supported by authorized health claims under the Nutrition and Health Claims Regulation. Ingredients are usually categorized as fiber or carbohydrates without suggesting functional benefits. The focus of regulatory approval is on toxicological safety and manufacturing quality rather than clinical efficacy. EFSA has denied most prebiotic claims, such as those for FOS, GOS, and inulin (beyond laxation), due to a lack of solid evidence linking changes in the microbiota to measurable health benefits. Only specific claims, such as inulin/oligofructose for bowel function (at ≥ 10 g/day), are permitted, while broader “prebiotic” claims remain unauthorized, leading to varying national guidelines. (Tuohy et al. 2024).

In India, regulatory oversight is governed by the Food Safety and Standards Authority of India (FSSAI). The use of prebiotics is limited to ingredients included in approved schedules, and labeling must comply with FSSAI-specified requirements, including quantitative declaration and adherence to authorized ingredient lists. Health claims must be supported by scientific evidence demonstrating safety and intended benefit (Deehan et al. 2024; Bhattacharjee et al. 2025). Brazil’s ANVISA found misleading labelling for galacto-oligosaccharides, resulting in fines and recalls, prompting calls for stricter, evidence-based regulations akin to those of the European Food Safety Authority. Oligosaccharides like 2’-fucosyllactose are now approved with age-based limits and a 24-month labeling transition, based on FOS/inulin as dietary fibers. In contrast, countries such as Switzerland, Japan, and Canada allow health claims for probiotics when supported by adequate scientific evidence under certain conditions. (Dronkers et al. 2018). Overall, regulatory and labelling frameworks for prebiotic oligosaccharides remain fragmented globally, highlighting the need for harmonized, evidence-based standards to ensure consumer protection, scientific credibility, and responsible health communication.

### Cost-effectiveness and scalability of oligosaccharide production

The cost-effectiveness and scalability of oligosaccharide production face challenges across downstream processing, substrate selection, enzymatic efficiency, and large-scale microbial synthesis. Downstream processing (DSP), which involves techniques such as ligand-exchange chromatography and nanofiltration, is costly and complex, requiring continuous process optimization. Expensive starting materials in microbial synthesis drive up costs, necessitating the development of efficient microbial catalysts. Enzyme costs, particularly for hydrolytic enzymes such as endoinulinase, can be reduced by using recombinant microorganisms and low-value plant sources. For instance, the development of recombinant Bacillus chitosanase from Escherichia coli has been shown to be cost-effective and efficient for large-scale production (Cheng et al. 2021 a).

Separation and purification of oligosaccharides from reaction mixtures, especially those using liquid acids, present additional efficiency challenges. Using EFLC-HILIC for faster and more efficient separation using liquid CO2 (Bennett and Olesik 2017). Scalability issues arise in microbial synthesis, where product degradation and byproduct formation complicate large-scale production. Bioreaction and metabolic engineering require optimization to ensure regio- and stereoselectivity and enhance enzyme activity (Crater and Lievense 2018). Agricultural waste utilization, particularly lignocellulosic waste, offers a sustainable solution but demands process improvements for large-scale viability (Cano et al. 2020). To overcome these challenges, optimizing DSP techniques, developing high-efficiency microbial strains, and utilizing agro-industrial by-products are essential. Advancements in enzyme technology and the integration of renewable resources can further enhance production efficiency, making oligosaccharides more cost-effective and scalable for functional food and therapeutic applications.

### Risks and potential adverse effects of oligosaccharide prebiotics

Although oligosaccharide prebiotics are widely recognized for their ability to modulate the gut microbiota and support host health, several limitations and potential adverse effects have been reported. One of the most frequently observed adverse outcomes is dose-dependent gastrointestinal discomfort, including bloating, flatulence, abdominal pain, and diarrhea. These effects primarily result from the rapid fermentation of oligosaccharides by intestinal microorganisms, leading to excessive gas production and osmotic shifts within the gut lumen. High intake levels, especially during the initial phases of supplementation, may exceed the adaptive capacity of the gut microbiota, resulting in transient but clinically relevant digestive disturbances (Dou et al. 2022).

Individuals with irritable bowel syndrome (IBS) and other functional gastrointestinal disorders represent a particularly sensitive population. Many oligosaccharide prebiotics fall under the category of fermentable oligo-, di-, monosaccharides and polyols (FODMAPs), which are known to exacerbate IBS symptoms such as bloating and abdominal pain. Accordingly, supplementation in these individuals should be approached with caution, emphasizing personalized dosing strategies and careful clinical monitoring (Wong 2016). Moreover, baseline microbiota composition strongly predicts responsiveness, with differences in dominant taxa (e.g., *Bacteroidaceae* versus *Bifidobacteriaceae*) influencing fermentation rates and short-chain fatty acid (SCFA) production. In IBS patients, prior microbiota profiles often necessitate dietary adaptation, such as a low-FODMAP phase, before prebiotic introduction (Hustoft et al. 2017).

Not all oligosaccharides uniformly support the growth of beneficial bacterial taxa. Certain *Lactobacillus* strains exhibit limited growth on plant-derived oligosaccharides compared with substrates such as human milk oligosaccharides, leading to variable or inconsistent responses across individuals. In the absence of sufficient beneficial microbes, prebiotics may inadvertently stimulate opportunistic or gas-producing bacteria (Zhang et al. 2022a). In addition, excessive fermentation by-product production, such as propionic acid, has been associated with adverse metabolic and neurobehavioral effects, including impaired glucose homeostasis, hyperglycemia, and anxiety-like behaviors (Divyashri et al. 2023).

Short-chain variants like FOS (DP 2–9) ferment quickly in the proximal colon, often causing bloating and gas. In contrast, longer-chain types (DP > 10, such as inulin) ferment more slowly and distally, potentially reducing discomfort but needing higher doses for effectiveness. (Zhao et al. 2024). Short-chain FOS up to 40 g/day are well-tolerated after adaptation, but initial high doses (> 10 g) can cause symptoms. Low doses of longer chains are less effective due to incomplete fermentation, requiring 5–20 g for Bifidobacteriaceae enrichment. (Lee et al. 2023).

Additional concerns relate to the quality and purity of commercial prebiotic formulations. Low-grade or impure products may disrupt balanced fermentation, reduce efficacy, or increase adverse gastrointestinal effects due to contaminants or uneven microbial stimulation (Zhang et al. 2018). For example, galactooligosaccharides (GOS) or FOS at doses above 6 g/day can promote bifidobacterial growth but may also increase bloating in sensitive individuals when purity and dosing are not adequately controlled (Urashima et al. 2025). Furthermore, the long-term safety of high-dose oligosaccharide supplementation remains insufficiently characterized. While short-term use is generally considered safe in healthy populations, prolonged consumption at supraphysiological levels may disrupt microbial equilibrium or interfere with nutrient absorption (Schönknecht et al. 2023).

## Future perspectives and emerging trends of oligosaccharides as prebiotics

### Role of artificial intelligence and bioinformatics in prebiotic research

AI is creating new opportunities in predictive models of microbial communication. The AI models demonstrate how groups of microbes in the gut cooperate and how they impact the host’s metabolism and immune system. Bioinformatics, such as omics, genomics, transcriptomics, epigenomics, proteomics, and metagenomics data, help in understanding the relationships and patterns, which are essential for prebiotic research by identifying the significant pathways and molecules. Utilizing AI models with bioinformatics helps design personalized therapies (Jamialahmadi et al. 2024; Patil et al. 2025; Al-Adham et al. 2025). AI has helped uncover important information by recognizing complex relationships between bacterial groups and human health using bioinformatics, which presents genomic and proteomic data. The experimental design for disease progression using microbiome traits as input to reinforcement learning algorithms offers targeted sampling by identifying microbial clusters and uncovering unseen trends through clustering and dimensionality reduction (Abavisani et al. 2024). The vast data gathered from microbiome investigations, artificial intelligence (AI), and machine learning (ML) aided in data analysis. By training on various microbiome and bioinformatics datasets, AI-based algorithms can identify patterns, correlations, and interactions that advance our knowledge of prebiotic development (Wani and Banday 2024). Together, AI-driven algorithms and bioinformatics may identify microbial variables associated with specific diseases by utilizing computational models with experimental data, demonstrate how microbial communities develop in response to external stimuli or interventions, and predict the effectiveness of a treatment based on the microbiome. This can reveal new links, generate hypotheses about how things function, and direct further research, opening the door to more specialized and personalized therapy (Li et al. 2022; Patil et al. 2025).

### Integration of prebiotics in personalized medicine and precision nutrition

Prebiotics customized to a patient’s particular Qmicrobiota can improve treatment outcomes and reduce adverse effects. AI is transforming personalized medicine by offering patients new options for medical care and treatment. Rapid advancements in AI are revolutionizing bioinformatics, enabling better disease diagnosis and potentially leading to the development of personalized treatment plans. Personalized medicine is more effective and targeted, and therapeutic strategies are enabled by AI models that mimic and predict pharmacological treatment outcomes, which have the desired properties in novel drug development (Jamialahmadi et al. 2024). Prebiotic therapies guided by AI can more precisely alter the gut microbiota by focusing on specific microbial indicators. AI-powered algorithms can quickly analyze intricate microbiome data and reveal functional genes that serve as significant markers of gut health and disease progression. Based on the unique microbiota profiles of each patient, prebiotic treatment can be customized to provide precision and personalized prebiotic medicine procedures, as well as enhanced disease-preventive tactics (Patil et al. 2025). With this strategy, the effectiveness of dietary treatments is increased by selecting prebiotics that support each person’s gut microbiome. A treatment option known as fecal microbiota transplantation (FMT) has been linked to intestinal, neurological, cardiovascular, and metabolic diseases. Understanding this can predict (through AI and bioinformatics application) the effective prebiotics that could regulate specific microbial communities in an individual and encourage their safety and patient compliance (Quaranta et al. 2022).

### Next-generation prebiotics: exploring novel sources and synthetic modifications

Future therapeutics to treat disease with next-generation prebiotics (NGPs) are gaining attention, enabled by molecular analysis technologies and high-throughput DNA sequencing. Prebiotics offer selective utilization of host microorganisms, conferring a health benefit that plays a significant role in NGPs. Researchers are working on NGPs to maintain their efficacy and manage to colonize the GIT to treat metabolic disorders (Fei et al. 2023). The health benefits of NGPs can directly stimulate the selective growth of bacteria or indirectly create a favorable environment for one bacterium over another. They will reduce the harmful effects of antibiotics, diarrhea, stress, and other drugs on gut microflora (Panesar et al. 2013). Omics technology, novel sources, innovative processing, and synthetic modifications are new, evolving fields in the NGPs to enhance functionality, protection, and health benefits.

Recent advancements in prebiotic production technologies help overcome the drawbacks of classical technologies, such as the adaptation of microbial enzymes in enzyme immobilization technologies, which promote polysaccharide hydrolysis in an efficient way to produce oligosaccharides with high yield and purity. Technologies like high hydrostatic pressure, nanotechnology, microwave irradiation, and ultrasound could play a significant role in future NGP production (Panesar et al. 2013; Ahmed and Goyal 2024). NGPs play a substantial role in supporting preventive health care and nutrition through a multifaceted approach. Polyphenols improve inflammation, metabolism, and immunity by promoting the growth of beneficial microorganisms through prebiotics and SCFAs. Because they are antibacterial and preserve the integrity of the gut-intestinal layer, SCFAs are advantageous to the host. NGPs are also focused on the protective effects of enhancing antioxidants. Subjecting GOS to fermentation by colonic bacteria into SCFAs helps reduce difficulties in hydrolysis by human digestive enzymes, improve intestinal Bifidobacterium, and reduce microecological fluctuations. Nutritional intervention with inulin helps treat metabolic disorders such as hepatic steatosis and obesity by improving the specific microbiome. Alginate-gelatin microgels containing *Lactobacillus salivarius* Li01 and *Pediococcus pentosaceus* Li05 retained their structures in gastric simulations, suggesting a possible protective effect on gut flora (Fei et al. 2023). FOS, inulin, and GOS are well-established prebiotics, whereas advancements in these established prebiotics could be considered NGPs. Raffinose, epilactose, and neoagaro-oligosaccharides are considered underdeveloped prebiotics, whereas XOS, COS, lactosucrose, and IMOS are emerging NGPs (Cardoso et al. 2021).

Recent advances in characterizing the microbiota have driven the development of low-cost sequencing techniques and bioinformatic tools for prebiotic production. Foodomics, an interdisciplinary approach that addresses health outcomes related to food consumption, the host metabolome, and the assembly of gut microbiota data, helps us understand how prebiotics affect our bodies and their mechanisms of action (Singh et al. 2024b). Metabolomics is a novel omics technology focusing on targeted and untargeted metabolites in samples to generate hypotheses and identify the cause of intervention (Lee Nen That et al., 2022). Sequencing technologies like Sanger sequencing detect predisposition to disease well before the genes are activated. Fast and cost-effective next-generation sequencing technologies, such as Roche 454, 16 S amplicon, and shotgun DNA sequencing, underscore the importance of identifying the sources of specific prebiotics (Fei et al. 2023). Bamboo shoot (basal part) dietary fiber is a novel prebiotic resource, with dynamic high-pressure microfluidization (DHPM) and enzymatic hydrolysis techniques used to improve solubility and modify texture, thereby enhancing prebiotic properties (Lee Nen That et al., 2022).

To replace synthetic compounds, the synthesis of biopolymer natural compounds from agro-wastes through emerging technologies, including microwave-assisted, ultrasound, and enzymatic hydrolysis, is a new development in the research and industrial sectors (Iñiguez-Moreno et al. 2024). Synthetic modifications, such as encapsulation and chemical changes, play essential roles in enhancing prebiotics’ health benefits and protective effects on gut microbiota, stability, and functionality. The biotransformation technology for encapsulated prebiotics, emerging methods that enhance antioxidant and prebiotic properties. Encapsulation made from protein and maltodextrin helps to deliver therapeutic effects on human inflammatory, cancer, and cardiovascular diseases (Bannikova et al. 2021). Chemical modifications, such as the substitution of -Br, -Cl, -CH3, and -NO2 groups onto natural flavonoids, modulate the gut microbiota by slowing harmful bacteria and promoting beneficial bacteria (Perz et al. 2024).

### Potential role in gut microbiome modulation for longevity and anti-ageing

In contrast to population and species diversity, the complex gut bacterial community is unstable and subject to age-related changes due to pathophysiological processes. The changes in gut microbiota can also be influenced by antibiotics, medications, and other factors (Bedani et al. 2016). Changes increase the risk of cardiometabolic, immunomodulatory, frailty, and neurodegenerative disease progression in older people due to increased gut pathogenic microorganisms. Consuming prebiotics improves the gut microbiota, providing anti-inflammatory effects, increasing intestinal barrier integrity, regulating nutrient-sensing pathways, protecting against age-related diseases, and optimizing mitochondrial function (Luo et al., 2024). Prebiotic therapy has been shown in multiple trials to alter the gut microbiota of older adults, promoting longevity and preventing aging (Barone et al. 2022). The selective utilization of prebiotics (specifically NGPs), as discussed, improves and protects the gut microbiota in age-related conditions. One research project on prebiotics reports the recovery of anti-aging microbiomes in elderly patients treated with prebiotics for 4 weeks. In a randomized, double-blind study, older patients with frailty who showed changes in their gut microbiota demonstrated improved gut microbiota and muscle strength after taking prebiotics (Ni Lochlainn et al. 2021; Wilms et al. 2021). SCFAs produced from the prebiotics enhance the gut barrier and modulate immune responses by controlling cytokine release and preventing endotoxemia (Liu et al. 2016).

## Future directions for developing oligosaccharide prebiotics

Oligosaccharide prebiotics have emerged as crucial bioactive compounds with significant implications for gut health, metabolic regulation, and immune modulation (Davani-Davari et al. 2019). Their ability to selectively stimulate the growth of beneficial gut bacteria, such as Bifidobacterium and Lactobacillus, underscores their role in enhancing digestive health and overall well-being. By promoting SCFA production, these prebiotics contribute to improved gut barrier function, reduced inflammation, and potential benefits for metabolic disorders such as obesity and diabetes (Bevilacqua et al. 2024). Additionally, their influence on the gut-brain axis opens new avenues for exploring their role in mental health and neurodegenerative disorders (Ansari et al. 2023).

The growing demand for functional foods has positioned oligosaccharide prebiotics as key ingredients in the food and pharmaceutical industries (Farias et al. 2019). Naturally sourced from plants such as chicory, garlic, and bananas, or synthesised through enzymatic and microbial processes, these prebiotics offer versatility in their applications (see Table 5). They improve the nutritional value of food products and enhance texture, stability, and taste, making them highly desirable for food formulation (Ferreira et al. 2023). Advances in microbiome research have further paved the way for personalised prebiotic therapies, in which tailored nutritional interventions could optimise gut microbiota composition based on individual health needs.

**Table 5:** Common prebiotics, their sources and structural details

Prebiotic	Source	Structure	Reference
Inulin	Chicory root, garlic, onion	Fructan (β(2→1) fructosyl units)	(Bandyopadhyay et al. 2021)
Fructooligosaccharides (FOS)	Carrots, Banana, onion	Short-chain fructans	(Guerra et al. 2023)
Galactooligosaccharides (GOS)	Human and cow milk	Galactose units linked by β-glycosidic bonds	(Marín-Manzano et al. 2020)
Xylooligosaccharides (XOS)	Corn cobs, bamboo, birchwood	β(1→4)-linked xylose units	(Gupta et al. 2022)
Lactulose	Synthetic (from lactose)	Disaccharide of galactose and fructose	(Delgado-Fernández et al. 2019)
Isomaltooligosaccharides (IMO)	Corn starch	Glucose polymers with α(1→6) and α(1→4) linkages	(Bommasamudram et al. 2023)
Pectic oligosaccharides	Apple peels, citrus fruits	Derived from pectin (polygalacturonic acid)	(Foti et al. 2022)
Arabinooligosaccharides	Sugar beet pulp	Arabinose units	(Martínez-Gómez et al. 2024)
Mannanoligosaccharides (MOS)	Yeast cell walls	Mannose polymers	(Cagliari et al. 2023)
Beta-glucans	Oats, barley, mushrooms	β(1→3), β(1→4) glucans	(Astiz et al. 2023)
Resistant starch	Green bananas, legumes	Starch not digested in the small intestine	(Kadyan et al. 2022)
Polydextrose	Synthetic (glucose polymer)	Randomly bonded glucose	(Thompson et al. 2024)
Raffinose	Legumes, whole grains	Galactose-glucose-fructose trisaccharide	(Kasprowicz-Potocka et al. 2022)
Stachyose	Beans, soybeans	Tetrasaccharide of two galactose, one glucose, one fructose	(Aulia H et al. 2024)
Cyclodextrins	Enzymatic conversion of starch	Cyclic oligosaccharides of glucose	(Liu et al. 2023)
Soy oligosaccharides	Soybeans	Raffinose and stachyose derivatives	(Rana et al. 2024)
Chitin oligosaccharides	Crustacean shells	N-acetylglucosamine units	(Ismail and Emran 2020)
Chitosan oligosaccharides	Deacetylated chitin	Glucosamine polymers	(Kim et al. 2024a)
Glucomannan	Konjac root	Glucose and mannose β(1→4) linked	(Li et al. 2021)
Levan	Bacteria, some plants	Fructan with β(2→6) linkages	(Cheng et al. 2021)
Agave inulin	Agave plant	Fructan	(Conceição Apolinário et al. 2020)
Jerusalem artichoke inulin	Jerusalem artichoke	Fructan	(Afoakwah and Mahunu 2022)
Milk oligosaccharides	Human milk	Fucosylated and sialylated oligosaccharides	(Mollova et al. 2023)
Maltooligosaccharides	Starch hydrolysates	Linear glucose polymers	(Cheon et al. 2023)
TOS (Transgalactooligosaccharides)	Fermented dairy products	Galactose-rich oligosaccharides	(Ambrogi et al. 2023)
Cereal beta-glucans	Oats, barley	β(1→3)(1→4) glucans	(Edo et al. 2024)
Feruloylated oligosaccharides	Cereal brans	Oligosaccharides with ferulic acid	(Younes and Karboune 2023)
Laminarin	Brown algae	β(1→3)-glucan	(Zhou et al. 2025)
Larch arabinogalactans	Larch tree	Arabinose and galactose polymers	(Sun et al. 2021)
Psyllium oligosaccharides	Psyllium husk	Polysaccharide hydrolysates	(Martellet et al. 2022)

Despite these promising benefits, several challenges hinder the widespread application of oligosaccharide prebiotics. Stability issues during food processing, optimal dosing strategies, and regulatory hurdles remain key concerns for researchers and industry stakeholders. Moreover, the cost-effectiveness of large-scale production and ensuring bioavailability in functional food matrices require further technological advancements. To address these challenges, innovative solutions such as synbiotic formulations (combining prebiotics with probiotics), nanotechnology-based delivery systems, and next-generation prebiotics are being explored to enhance efficacy and sustainability.

Future research should expand our understanding of the precise mechanisms through which oligosaccharide prebiotics exert their health benefits. Clinical studies validating their long-term safety and efficacy will be crucial for regulatory approvals and consumer acceptance. Integrating artificial intelligence and bioinformatics in prebiotic research could also accelerate the discovery of novel oligosaccharides with enhanced functional properties. Additionally, precision nutrition, where prebiotics are tailored to individual microbiome profiles, holds great promise for advancing personalised healthcare solutions. As the global market for prebiotic-enriched functional foods continues to expand, collaboration between researchers, food manufacturers, and policymakers will be essential to bridge the gap between scientific advancements and practical applications. Ensuring that oligosaccharide prebiotics meet regulatory standards while maintaining cost-effectiveness and scalability will determine their long-term success in the industry. Furthermore, public awareness initiatives highlighting the health benefits of prebiotics will drive consumer acceptance and promote their incorporation into daily diets.

##  Conclusion

Oligosaccharide prebiotics represent a powerful tool in modern nutrition and therapeutic strategies. Their role in gut health, immune function, and metabolic regulation positions them at the forefront of preventive healthcare. While challenges persist, ongoing research and technological advancements are set to revolutionise their applications in functional foods and therapeutics. By addressing existing limitations and leveraging emerging innovations, oligosaccharide prebiotics have the potential to make a significant contribution to global health and wellness in the coming decades.

**Graphical Abstract**.

## Abbreviations

“2′-FL: 2′-Fucosyllactose
3FL: 3-Fucosyllactose
5-FU: 5-Fluorouracil
5-HT: Serotonin
5-HTP: 5-Hydroxytryptophan
AD: Alzheimer’s disease
AhR: Aryl hydrocarbon receptor
AI: Artificial intelligence
AMPK: AMP-activated protein kinase
AOS: Acidic/Alginate oligosaccharides
Aβ: Amyloid-beta
BDNF: Brain-derived neurotrophic factor
CBPs: Carbohydrate-based prebiotics
CD: Cyclodextrins
CE: COS-EGCG conjugate
CO_2_: Carbon dioxide
COS: Chitosan/Carrageenan oligosaccharides
COS-Se: Selenium-conjugated chitosan oligosaccharides
CRISPR: Clustered regularly interspaced short palindromic repeats
DC: Dendritic cells
DHPM: Dynamic high-pressure micro-fluidization
DP: Degree of polymerization
DSP: Downstream processing
DSS: Dextran sulfate sodium
DTH: Delayed-type hypersensi
EAAU: Experimental autoimmune anterior uveitis
EFLC-HILIC: Enhanced fluidity liquid chromatography–hydrophilic interaction liquid chromatography
EFSA: European Food Safety Authority
EGCG: Epigallocatechin gallate
EGFR: Epidermal growth factor receptor
EU: European Union
FAS: Fatty acid synthase
FDA: Food and Drug Administration
FODMAPs: Fermentable oligo-, di-, monosaccharides and polyols
FMT: Fecal microbiota transplantation
FOS: Fructooligosaccharides
FSANZ: Food Standards Australia New Zealand
FTC: Federal Trade Commission
FXR: Farnesoid X receptor
GDM: Gestational diabetes mellitus
GIT: Gastrointestinal tract
GOS: Galactooligosaccharides
GPCRs: G-protein-coupled receptors
GPR41/GPR43: G-protein-coupled receptor 41/43
GSK3β: Glycogen synthase kinase-3 beta
HA: Hyaluronic acid
HDACs: Histone deacetylases
HFD: High-fat diet
HFHF: High-fat high-fructose diet
HMOs: Human milk oligosaccharides
HOMA-IR: Homeostatic model assessment of insulin resistance
HPA axis: Hypothalamic-pituitary-adrenal axis
IAA: Indole-3-acetic acid
IBD: Inflammatory bowel disease
IBS/IBS-D: Irritable bowel syndrome/IBS with diarrhea
IF: Intermittent fasting
IL-1β/IL-6/IL-10/IL-13/IL-17A/IL-22: Interleukins
IMOS/IMO: Isomaltooligosaccharides
IPA: Indole-3-propionic acid
IR: Insulin resistance
IRS-1: Insulin receptor substrate-1
ISAPP: International Scientific Association for Probiotics and Prebiotics
KMOS: Konjac mannan oligosaccharides
KOGM: Konjac oligo-glucomannan
LFD: Low fermentable diet
LPA: Laminaripentaose
MAOS: Mannan-derived oligosaccharides
MAN1A1: Mannosidase alpha class 1A member 1
MAPK: Mitogen-activated protein kinase
ML: Machine learning
MMP-9: Matrix metalloproteinase-9
MOS: Mannan oligosaccharides
MOO: Morinda officinalis oligosaccharides
MP: Macrophages
NAFLD: Non-alcoholic fatty liver disease
NF-κB: Nuclear factor kappa-B
NGPs: Next-generation prebiotics
NK cells: Natural killer cells
NLRP3: NOD-like receptor family pyrin domain containing 3
NTDs: Neural tube defects
OA: Osteoarthritis
OOJ: Ophiopogon japonicus oligosaccharides
PD-1/PD-L1: Programmed cell death protein-1/ligand-1
PGC-1α: Peroxisome proliferator-activated receptor gamma coactivator-1 alpha
PI3K/Akt: Phosphoinositide 3-kinase/Protein kinase B
PM: Polymannuronic acid
POS: Pectic oligosaccharides
PRDM16: PR domain containing 16
RS: Resistant starch
SCFAs: Short-chain fatty acids
SCORAD: Scoring Atopic Dermatitis index
scFOS: Short-chain fructo-oligosaccharides
Sirt-1: Sirtuin-1
sIgA: Secretory immunoglobulin A
SLE: Systemic lupus erythematosus
SS NPs: Solid-state nanoparticles
T2DM: T2DMType 2 diabetes mellitus
TDA: Traditional dietary advice
Th1/Th2: T helper type 1/type 2 cells
TLR2/TLR4: Toll-like receptor 2/4
TNBC: Triple-negative breast cancer
TNBS: Trinitrobenzene sulfonic acid
TNF-α: Tumor necrosis factor-alpha
Treg: Regulatory T cells
TXNIP: Thioredoxin-interacting protein
UCP1: Uncoupling protein-1
US: United States
VEGF: Vascular endothelial growth factor
VPA: Valproic acid
WP: Wheat peptides
XOS: Xylooligosaccharides
ZO-1: Zonula occludens-1
